# Evaluation of Cardioprotective Effect of 3,5,3′-Tri-iodo-L-thyronine in Isoproterenol-Induced Cardiotoxicity

**DOI:** 10.1155/2011/908367

**Published:** 2011-11-24

**Authors:** Vinay Mishra, Priya Ghumatkar, Maulik V. Patel, Shilpesh Devada, Ramchandra Ranvir, Prabodha Swain, Rajesh Sundar, Rajesh Bahekar, Mukul R. Jain

**Affiliations:** ^1^Department of Pharmacology and Toxicology, Zydus Research Center, Sarkhej-Bavla N.H. No. 8A, Moraiya, Ahmedabad 382213, India; ^2^Department of Molecular Pharmacology, Zydus Research Center, Sarkhej-Bavla N.H. No. 8A, Moraiya, Ahmedabad 382213, India; ^3^Department of Medicinal Chemistry, Zydus Research Center, Sarkhej-Bavla N.H. No. 8A, Moraiya, Ahmedabad 382213, India

## Abstract

T_3_ (3,5,3′-triiodothyronine) has drawn relatively little attention in relation to cardiovascular (CVS) diseases. The present study was designed to evaluate the cardioprotective action of T_3_ in isoproterenol-(ISO-) induced cardiac toxicity. Female Wistar rats were exposed with ISO (100 mg/kg, body weight, subcutaneously) for 2 days at the interval of 24 h followed by T_3_ (3 **μ**g/kg, body weight, orally) treatment for 3 days. Positive control rats received only ISO (100 mg/kg, body weight, subcutaneously) for 2 days at the interval of 24 hrs. Control group animals received normal saline as a vehicle. As expected, ISO-induced significant changes were observed in low-density lipoprotein, total cholesterol, ALT, CK-MB to TCK ratio, and prolongation of QT interval in electrocardiogram, which is toward normalization after T_3_ treatment. Lower heart weight, upregulation of cardiac myosin heavy chain alpha (MHC-**α**), and reduced inflammatory cell infiltration, myonecrosis, vacuolar changes, and a trend toward normal cardiac muscle fiber architecture in microscopic examination of cardiac tissue further support the cardioprotective effect of T_3_.

## 1. Introduction

In the past decades, a different class of drugs has been used for the control of myocardial ischemia and associated pathologies, but thyroid hormone has attracted relatively little attention in relation to CVS diseases. Heart ischemia is one of the main causes related to sudden death in the world. It is believed that exogenous supply of T_3_ causes increase in heart rate, in myocardial infarction leading to higher mortality rate and low T_3_ levels have cardioprotective action in heart diseases. Recent data has shown that low levels of triiodothyronine (T_3_) in case of heart failure are often associated with increased mortality and morbidity [1–3]. The interaction between thyroid hormone and heart indicates that T_3_ levels as well as clinical severity may be correlated with myocardial contractility in the patient with stress cardiomyopathy [4]. It is well known that ISO-induced cardiotoxicity in rat models is a widely used for evaluation of cardioprotective effect of various drugs. ISO causes severe stress in the myocardial tissue of animals resulting in myocardial infarction in animals due to the action on the sarcolemmal membrane, stimulation of adenylate cyclase, activation of Na^+^ and Ca^2+^ channels, and exaggerated Ca^2+^ inflow and energy consumption leading to cellular death. Additionally, free radicals produced by ISO could initiate peroxidation of membrane bound polyunsaturated fatty acids, leading to both functional and structural myocardial injuries [5]. Therefore, a cardioprotective effect of T_3_ needs to be demonstrated clearly in animal models.

With these objectives in mind, developing new therapeutic strategies to treat specific cardiac diseases through thyroid hormones can be an added advantage in area of medical research.

## 2. Materials and Methods

### 2.1. Experimental Animals

Healthy young nulliparous female Wistar rats 5–7 weeks (120 ± 15 g), obtained from Animal Research Facility of Zydus Research Centre, Ahmedabad, were housed in IVC (Individually Ventilated Cage) under standard laboratory conditions: temperature (25 ± 3°C), relative humidity (30 to 70%), and photoperiod (light and dark cycle of 12 h each) with food and water provided *ad libitum*. Protocol for this study was approved by Institutional Animal Ethics Committee (IAEC).

### 2.2. Experimental Design

Female rats were randomized into four groups comprising of five animals in each group.

**Group I**

Control rats received normal saline subcutaneously for first 2 days and distilled water remaining 3 days via gastric intubation.

**Group II**

Rats received ISO in normal saline subcutaneously (100 mg/kg b·wt/day), at an interval of 24 h for two days.

**Group III**

Rats received T_3_ (3 *μ*g/kg b·wt/day) orally by gavage for 3 consecutive days after 2 days ISO treatment.

**Group IV**

Rats treated with T_3_ alone (3 *μ*g/kg b·wt/day) via gastric intubation for 5 consecutive days.

Extra set of animals comprising five animals in each groups were used for electrocardiogram.

### 2.3. Dose Selection

A pilot study was conducted to establish the optimum dose of the T_3_ and its duration of treatment which exhibits maximum cardioprotective effect. Rats were treated with T_3_ (1, 3, 5, 8 and 15 *μ*g/kg/day, p.o.) of duration 3, 7, and 14 days after 2 days treatment with isoproterenol. At the end of treatment, serum markers such as Alanine aminotransferase (U/L), Alkaline phosphatase (U/L), Aspartate aminotransferase (U/L), Creatine Kinase (U/L), and CKMB (IU/L) were evaluated. T_3_ (3 *μ*g/kg/day, p.o.) of duration 3 days was found to be most effective in reverting the biochemical alteration induced by Isoproterenol. In addition, exploratory study of Isoproterenol was also carried out (60, 80, 100, 150, and 200 mg/kg/day, s.c.), dose of 100 mg/kg was selected based on high mortality at 150 and 200 mg/kg, and clear cut serum cardiac marker changes at 100 mg/kg.

### 2.4. Test Chemical

3,5,3′-triiodo-L-thyronine (T_3_) [Sigma-Aldrich, USA], Isoprenaline hydrochloride, [Sigma-Aldrich Chemical Pvt. Ltd., USA], Polyoxyethylene sorbitan monooleate (Tween 80) [Merck specialities private limited, Bombay, India], Dimethyl sulphoxide (DMSO) [Qualigens fine chemicals GlaxoSmithkLine Pharma Limited, Bombay, India], TRIzol reagent [Invitrogen, Life Technologies, Carsbad, CA, USA].

### 2.5. Observations

#### 2.5.1. Electrocardiogram

Electrocardiogram was measured at the end of treatment period (Day 6). The electrocardiographic patterns were recorded by three lead-sixteen channel polygraph (Biopac Systems Inc., USA). Parameters evaluated were PQ, QT, RR, and QRS interval. Female Wistar rats were anaesthetized with ketamine and xylazine at a dose of 70 and 7 mg/kg b·wt, respectively, by intraperitoneal route for ECG measurement.

### 2.6. Clinical Pathology

Blood samples were collected from retro-orbital plexus for serum biochemistry and hormonal profile at the end of treatment to correlate results of ECG, histopathology, and gene expressions studies.

#### 2.6.1. Serum Biochemistry

Biochemical analysis was done using Daytona autoanalyser (Randox Laboratories, UK). Details of analytes evaluated and the methods used are as follows: total cholesterol (mg/dL) (TC-cholesterol oxidase), low-density lipoprotein (mg/dL) (LDL-Direct clearance), alanine aminotransferase (U/L) (ALT-UV without p5p), alkaline phosphatase (ALP-PNP AMP buffer), aspartate aminotransferase (U/L) (AST-UV), calcium (mg/dL) (Arsenazo), creatine kinase (U/L) (CK-UV: NAC activated), total T_3_ (TT_3_), and total thyroxine (TT_4_) were estimated by ELISA method (CL, Biotech), CKMB (IU/L) (ELISA method CL, Biotech).

### 2.7. Histopathology

At terminal necropsy (day 6), animals were humanely euthanised by carbon dioxide asphyxiation. Animals were subjected to complete gross examination. Weight of the heart was estimated and fixed in 10% formal saline. Paraffin sections were prepared and stained with hematoxylin-eosin for histopathological examination. Massion's Trichrome special stain was done for fibroblast.

### 2.8. Gene Expression

Tissue samples from heart were dissected and snap-frozen in liquid nitrogen immediately at terminal necropsy and stored at −70 ± 2°C for further analysis. Equal amount of frozen heart tissue and TRIZOL reagent (1 mL/100 mg of tissue) was homogenized and total RNA (Ribonucleic acid) was isolated. Quantitation of total RNA was performed using Biophotometer (Eppendorf, Germany), and the quality of RNA was ascertained by agarose gel electrophoresis. For gene expression of MHC-alpha, Myh6F-(CACCCTGGAGGACCAGATTA) and Myh6R-(TGGATCCTGATGAACTTCCC) specific RT-PCR (Real Time-Polymerase Chain Reaction) primers were used. First-strand cDNA (Complementary deoxyribonucleic acid) synthesis was achieved with 2 *μ*g of total RNA in a final volume of 20 *μ*L. About 2 *μ*L from this reaction cocktail was used directly to conduct PCR amplification in presence of SYBR-Green following real-time RT-PCR using ABI-7300 system (Applied Biosystem, Singapore). SYBR Green-based real time RT-PCR was used to estimate the levels of transcripts (MHC-alpha) expressed in heart samples collected from experimental groups.

### 2.9. Statistical Analysis

Statistical analysis was performed using GraphPad Prism Version 4.00. Data was analyzed for statistical significance by dose-wise by using GraphPad Prism Version 4.00. Numerical results were processed to get group mean and standard deviation. ANOVA (Analysis of Variance) was used for the comparison of different dose groups with the control and ISO-treated group. Post hoc test employed to analyze data after ANOVA was Dunnett's test (parametric). Analysis of data was done at 1% and 5% level of significance.

## 3. Results

### 3.1. Electrocardiogram

Morphological evaluation of electrocardiogram revealed marked changes in ST segment and T wave. ISO-(100 mg/kg) treated groups showed marked T-wave depression and complete T-wave inversion. Significant prolonged QT interval and decrease QRS interval was found in ISO-(100 mg/kg) treated group, whereas the combination group (ISO + T_3_) showed normalization trend of T-waves, QT interval, and QRS interval. Heart rate was increased in ISO injected rats compared to control rats and was found nonsignificant. T_3_-treated group alone was comparable with control group (Figure 5) (Table 1).

### 3.2. Lipid Profile

**Figure 1:**
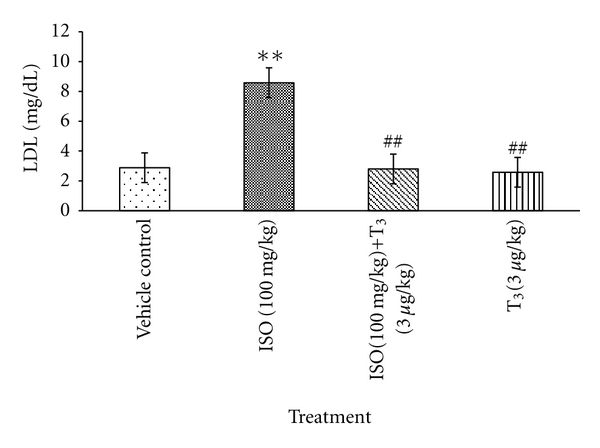
Effect of T_3_ on low-density lipoprotein, values are presented as Mean ± SD, *n* = 5, **: significant from control group at 1% level (*P* < 0.01), ^##^: significant from ISO control at 1% level (*P* < 0.01), SD: Standard Deviation.

LDL level was significantly increased by 197.9% (**P < 0.01) in ISO-treated rats in comparison with control rats. Treatment with T_3_ in ISO-injected rats (T_3_ + ISO) showed significant low level of LDL by 67.4% (^##^ *P* < 0.01), which was observed in comparison with ISO treated rats (Figure 1). A significant (*P < 0.05) elevation in total cholesterol was observed in ISO administered animals in comparison to control group which was reduced after (T_3_ + ISO) treatment.

### 3.3. Cardiac Injury Marker

**Figure 2:**
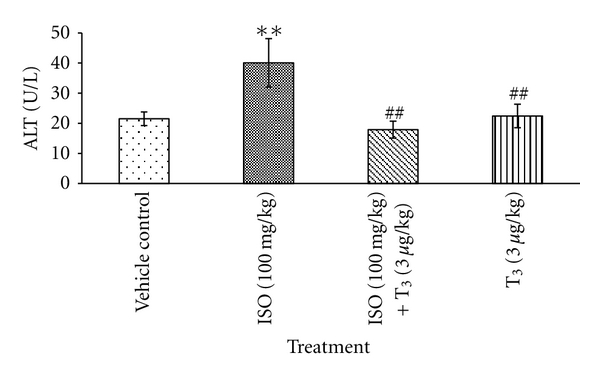
Effect of T_3_ on ALT, values are presented as Mean ± SD, *n* = 5, **: significant from control group at 1% level (*P* < 0.01), ^##^: significant from ISO control at 1% level (*P* < 0.01), SD: Standard Deviation.

**Figure 3:**
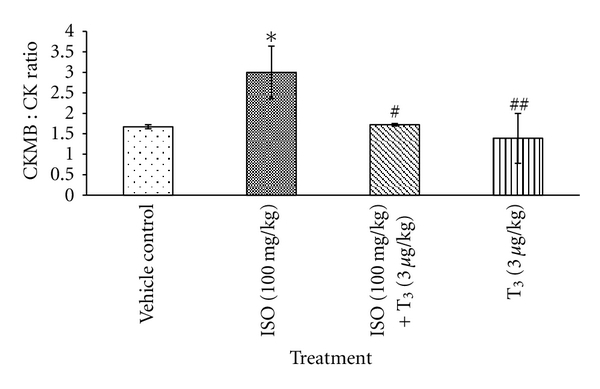
Effect of T_3_ on CKMB: CK Ratio, values are presented as Mean ± SD, *n* = 5, *: significant from control group at 0.05% level (*P* < 0.05), ^#^: significant from ISO control group at 0.05% level (*P* < 0.05), ^##^: significant from ISO control at 1% level (*P* < 0.01), SD: Standard Deviation.

Approximately, 2-fold significant elevation in ALT (**P < 0.01) was noticed in ISO-treated rats in comparison with control rats. Treatment with T_3_ in ISO-injected rats shown a significant decline in ALT (^##^ *P* < 0.01), in comparison with ISO-treated rats (Figure 2). T_3_ alone treated rats showed a significant decrease in ALT level in comparison with ISO-treated rats. CKMB: CK ratio was found to be significantly higher by 79.6% (*P < 0.05) in ISO-treated animals, which was significantly reduced after T_3_ + ISO treatment (Figure 3).

### 3.4. Hormonal Profile

Significant decrease in total thyroxine was noticed in all groups in comparison with control groups. However, no changes were observed in total T_3_ levels (Table 2).

### 3.5. Heart to Body Weight Ratio

**Figure 4:**
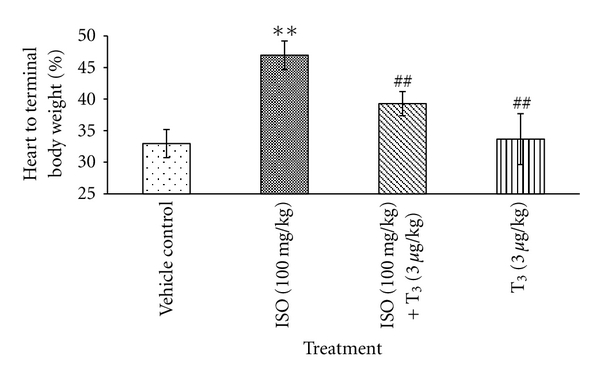
Effect of T_3_ on heart weight. Values are presented as Mean ± SD, *n* = 5, **: significant from control group at 1% level (*P* < 0.05), ^##^: significant from ISO control group at 1% level (*P* < 0.05), SD: Standard Deviation.

**Figure 5:**
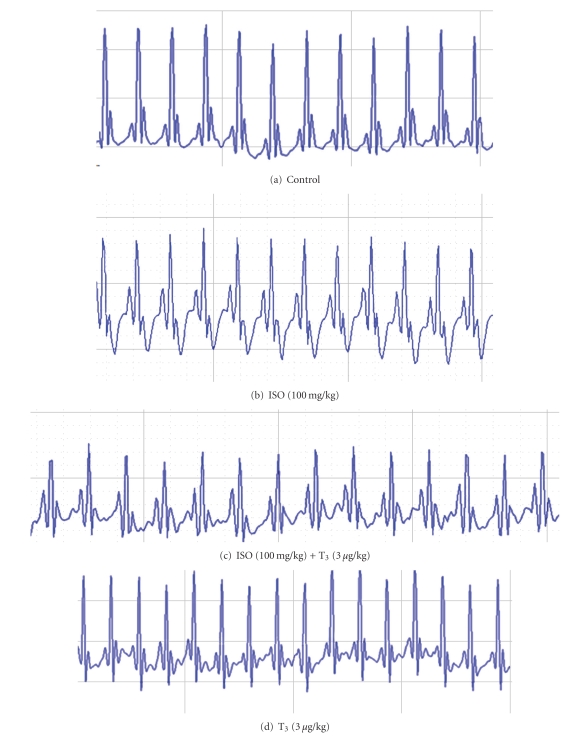
Electrocardiograms.

Relative heart weight was found significantly increased by 42.5% (**P < 0.01) in ISO-treated rat in comparison with control rats. After treatment with T_3_ in ISO-injected rats, heart weight was significantly reduced by 19.21% (^#^ *P* < 0.05), in comparison with ISO-treated rats (Figure 4).

### 3.6. MHC-*α* Gene Expression

**Figure 6:**
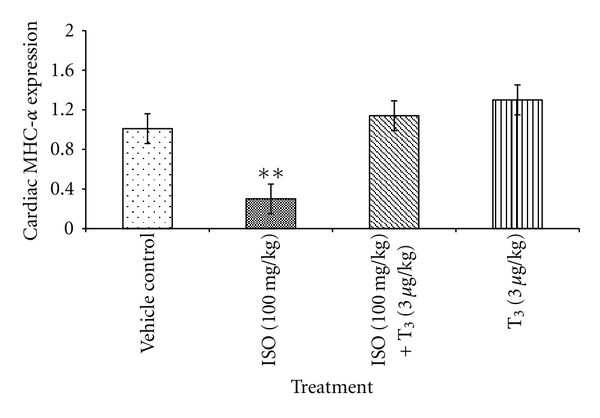
Effect of T_3_ on relative expression of MHC-*α*. Values are presented as Mean ± SD, *n* = 5, **: significant from control group at 1% level (*P* < 0.01), SD: Standard Deviation.

Significant downregulation of MHC-*α* expression was seen by 70.3% (*P < 0.05) in ISO-treated rats in comparison with control rats. T_3_ in combination with ISO and T_3_ treatment alone revealed marked upregulation of MHC-*α* in comparison with only ISO-treated rats (Figure 6). This upregulation of MHC-*α* was comparable with control animals.

### 3.7. Histopathological Findings in Heart

**Figure 7:**
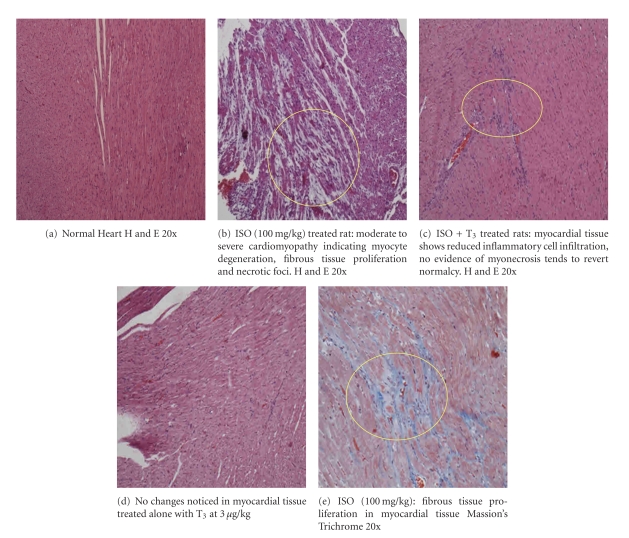
Histopathology of cardiac tissue.

Microscopic examination of heart tissue revealed moderate to severe cardiomyopathy in ISO-treated group (5/5). There was extensive myocyte membrane damage, myo-necrosis, proliferation of fibroblast, and infilration of mononuclear cells in ISO-treated group. In ISO + T_3_, group revealed minimal damage to the myocardium with much reduced myonecrosis, edema, and lymphocytic infilration in comparison with ISO group. No changes were noticed in control and T_3_ alone treated rats (Figure 7). Confirmation of proliferation of fibroblast was done with Massion Trichome.

## 4. Discussion

Lipid profile plays an important role in the pathogenesis of cardiovascular diseases, not only by way of hyperlipidemia and development of atherosclerosis, but also by modification of composition, structure, and stability of the cellular membranes. ISO markedly raises the low-density lipoprotein and total cholesterol levels [6]. A strong positive correlation has been documented between the risk of developing ischemic heart disease and serum LDL level [7]. Posttreatment with T_3_ successfully restored the elevated LDL-cholesterol and total cholesterol levels in the treatment group. These effects of T_3_ may be due to rapid increase in hepatic LDL receptor mRNA to promote the LDL clearance process [8, 9]. These alterations by T_3_ indicate the well-known physiological actions of T_3_ on lipid metabolism.

CK-MB isoenzyme activity is useful for both early diagnosis of myocardial infarction and other types of myocardial injury. CK-MB, TCK, AST, ALT, and ALP, which serve as the diagnostic markers, leak out from the damaged tissue to blood stream when cell membrane becomes more permeable or when rupture [10]. Sometimes serum CK-MB may raise as a result of noncardiac muscle damage, so the ratio of CK-MB to TCK is preferred over CK-MB alone. In the present study, ISO injected Wistar female rats showed significant elevation in the levels of ALT and CK-MB to TCK ratio, which were corroboration with the previous reports and findings of ISO such as necrotic damage of the myocardium and leakiness of the plasma membrane. T_3_ posttreatment resulted in the lowered activity of the marker enzymes in serum. Our observations demonstrated that T_3_ could maintain membrane integrity, thereby restricting the leakage of these enzymes [11].

ISO administration in rats showed treatment-related adverse effects in electrocardiogram. There was a significant prolongation in QT interval and decrease QRS interval. These changes may be due to consecutive loss of cell membrane in injured myocardium [12]. Changes in T-waves morphology in ISO treated animals indicates myocardial edema as a result of loss of cell membrane function [13]. These abnormal changes in ECG were not noticed in animals treated with T_3_ after ISO administration suggestive of the protective cell membrane role of T_3_. 

Exogenous administration of T_3_ has negative feedback suppressive effects on endogenous thyroxine levels than control rats. It is interesting to note that ISO-treated rats showed significant reduction in total thyroxine level in comparison with control group. This indicates that low thyroxine level may be responsible for cardiac adverse effects, which is further supported by our findings with experimental and clinical evidence. Low thyroid hormone states in heart failure are often associated with increased mortality and morbidity [2].

The relative expression of *α*- and *β*-MHC isoforms is altered in diseased state such as cardiac hypertrophy or failure where a shift from the normally predominant *α*-MHC toward *β*-MHC is observed [14, 15]. As a result, upregulation of *β*-MHC transcription is considered as an early and sensitive marker of cardiac hypertrophy [16]. *β*-MHC is characterized by lower filament sliding velocity but has a higher economy of energy consumption than *α*-MHC [17, 18]. This suggests that a shift from *α* to *β*-MHC might be an adaptive response in order to preserve energy. Studies on transgenic mice expressing predominantly *β*-MHC showed reduced mechanical function suggesting increased *β*-MHC expression which may have a detrimental effect on heart failure [19]. Increased *β*-MHC indicates the underlying adverse cardiac remodelling.

T_3_ treatment effectively prevented the relative shift in isoforms, from *α*-MHC to *β*-MHC, and may contribute to its beneficial effect in cardiac hypertrophy. It might be due to inhibition of the remodelling process by T_3_. Cardiac tissue of ISO-treated rats showed significantly downregulation of MHC-*α* gene which indirectly indicates the upregulation MHC-*β* gene and may be responsible for deleterious effect in heart. T_3_ increased MHC-*α* synthesis by increasing the DNA-dependent RNA synthesis and decreased MHC-*β* synthesis by inhibiting the DNA-dependent RNA synthetase enzyme.

Following ISO administration, significant higher weight of heart in comparison with control was noticed, which was significantly low in T_3_ + ISO-treated rats. Higher heart weight might be attributed to increased water content, edematous intramuscular space [20], and increased protein content. Posttreatment of T_3_ does not alter heart weight to longer extent indicative of its protective role on myocardium against mucopolysaccharides and cellular infiltration and thus preventing intramuscular edema. This may be further correlated with inhibition of relative shift from *α*-MHC to *β*-MHC by T_3_ and indicates its beneficial effect in preventing cardiac hypertrophy [21]. It is interesting to note that no change in heart weight was observed in T_3_-treated alone group rats at 3 *μ*g/kg.

Histopathological examination of cardiac tissue revealed moderate to severe cardiomyopathy which includes myocyte degeneration, inflammatory cell infiltration, fibrous tissue proliferation, and necrotic foci in ISO-treated animals. T_3_ in combination of ISO showed minimal inflammatory cell infiltration and trend toward normal cardiac muscle fiber architecture further confirmed the cardio protective effect of T_3_.

## 5. Conclusion

Administration of T_3_ hormone reveals the cardioprotective role from Isoproterenol-induced myocardial infarction in Wistar rats in this study. This was evident from the reversal of serum enzymes, ECG, and MHC gene expression profile and a trend towards normalization of cardiac muscle fiber architecture in histopathological evaluation.

## Figures and Tables

**Table 1:** Effect of T_3_ on electrocardiogram parameters in ISO-induced cardiac changes.

Group/Dose	(I)/Vehicle control	(II)/ISO (100 mg/kg)	(III)/ISO + T_3_ (3 *μ*g/kg)	(IV)/T_3_ (3 *μ*g/kg)
QT interval (ms)	93.33 ± 5.77	146.67 ± 15.28**	100.00 ± 14.52^##^	86.33 ± 3.51^##^
QRS interval (ms)	40.00 ± 4.15	30.33 ± 3.55*	43.33 ± 5.77	39.00 ± 3.61

Values are presented as Mean ± SD, *n* = 5, *P < 0.05 versus control, **P < 0.01 versus control, ^#^ *P* < 0.05 versus ISO (100 mg/kg), ^##^ *P* < 0.01 versus ISO (100 mg/kg), SD: Standard Deviation, ECG parameters are expressed in milisecond.

**Table 2:** Summary of hormonal profile.

Parameter	(I)/Vehicle control	(II)/ISO (100 mg/kg)	(III)/ISO + T_3_ (3 *μ*g/kg)	(IV)/T_3_ (3 *μ*g/kg)
Total T_3_ (ng/mL)	1.17 ± 0.13	1.08 ± 0.07	1.10 ± 0.02	1.06 ± 0.08
Total T_4_ (*μ*g/mL)	5.35 ± 1.23	2.80 ± 0.53*	2.81 ± 0.40**	1.95 ± 0.55**

Values are presented as Mean ± SD,  *n* = 5, *P < 0.05 versus control, **P < 0.01 versus control, ^#^ *P* < 0.05 versus ISO (100 mg/kg), ^##^ *P* < 0.01 versus ISO (100 mg/kg), SD: Standard Deviation.
